# Mammalian egg coat modifications and the block to polyspermy

**DOI:** 10.1002/mrd.23320

**Published:** 2020-01-31

**Authors:** Eileen Fahrenkamp, Blanca Algarra, Luca Jovine

**Affiliations:** ^1^ Department of Biosciences and Nutrition & Center for Innovative Medicine Karolinska Institutet Huddinge Sweden

**Keywords:** block to polyspermy, egg coat, fertilization, hardening, zona pellucida

## Abstract

Fertilization by more than one sperm causes polyploidy, a condition that is generally lethal to the embryo in the majority of animal species. To prevent this occurrence, eggs have developed a series of mechanisms that block polyspermy at the level of the plasma membrane or their extracellular coat. In this review, we first introduce the mammalian egg coat, the zona pellucida (ZP), and summarize what is currently known about its composition, structure, and biological functions. We then describe how this specialized extracellular matrix is modified by the contents of cortical granules (CG), secretory organelles that are exocytosed by the egg after gamete fusion. This process releases proteases, glycosidases, lectins and zinc onto the ZP, resulting in a series of changes in the properties of the egg coat that are collectively referred to as hardening. By drawing parallels with comparable modifications of the vitelline envelope of nonmammalian eggs, we discuss how CG‐dependent modifications of the ZP are thought to contribute to the block to polyspermy. Moreover, we argue for the importance of obtaining more information on the architecture of the ZP, as well as systematically investigating the many facets of ZP hardening.

## Graphical abstract

Post‐fertilization modifications of the zona pellucida (ZP) alter its properties and impact its ability to interact with sperm. In this review, we discuss how the contents of the exocytosed cortical granules may induce ZP hardening and establish the egg coat block to polyspermy.

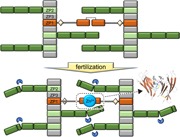

## 1 EGG COAT COMPOSITION, STRUCTURE, AND FUNCTION

The mammalian ZP is composed of three to four glycoprotein subunits, called ZP1–4 (Litscher & Wassarman, 2014). ZP4 is a pseudogene in mice, while it is expressed in humans (Conner, Lefièvre, Hughes, & Barratt, 2005; Goudet, Mugnier, Callebaut, & Monget, 2008; Hughes & Barratt, 1999; Lefièvre et al., 2004). Different species, on the contrary, lack other egg coat components: for example, in pig, bovine, and dog the ZP contains ZP4 but lacks ZP1 (Goudet et al., 2008; Hedrick & Wardrip, 1987; Noguchi et al., 1994; Topper et al., 1997). An even more complex picture develops if one also considers the vitelline envelope (VE) of other vertebrates, an extracellular matrix that corresponds to the mammalian ZP. This can contain additional subunits that are structurally related to ZP1–4, such as for example ZPY in *Xenopus*, ZPD in chicken, and ZPAX in amphibian and bird (Frankenberg & Renfree, 2018; Goudet et al., 2008; Lindsay, Wallace, & Hedrick, 2001; Nishio, Okumura, & Matsuda, 2018); on the contrary, in most species of fish, the VE lacks ZP2 (Litscher & Wassarman, 2018). In the case of chicken, the VE subunit composition also varies during oocyte development, so that ZP2 remains concentrated around the small germinal disk region of the mature egg (Nishio et al., 2014, 2018). Moreover, an apparently functional ZPAX orthologue was recently detected in the genome of a marsupial species (Frankenberg & Renfree, 2018). On the basis of this data, it can be concluded that the molecular composition of vertebrate egg coats is relatively species‐specific and that, with the exception of ZP3, the absence of individual components appears to be compensable. Notably, the biosynthesis of egg coats also varies significantly among vertebrate species, both in terms of where subunits are synthesized and the kinetics of their incorporation. However, it was conclusively established that mouse ZP components are coordinately synthesized over the course of 2–3 weeks by the growing oocyte (Wassarman & Litscher, 2018), whereas granulosa cells may possibly also contribute to the synthesis of human ZP subunits (Gupta, 2018).

**Figure 1:**
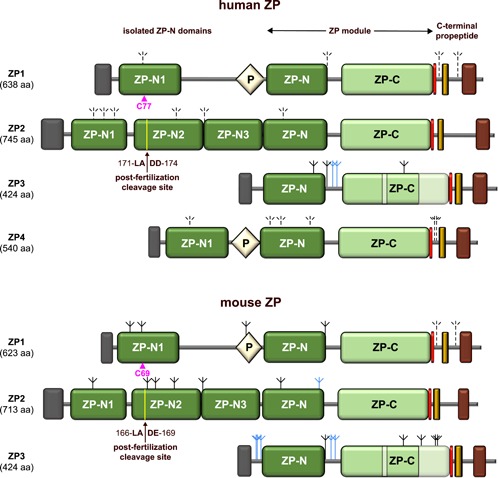
Scheme of the domain architecture of human and mouse ZP components. The positions of the conserved ZP1‐specific Cys essential for filament cross‐linking (Nishimura et al., 2019) and the post‐fertilization cleavage site of ZP2 important for ZP hardening (Gahlay, Gauthier, Baibakov, Epifano, & Dean, 2010) are marked by magenta arrowheads and black arrows, respectively. Dark gray rectangle, SP; light yellow diamond, trefoil/P domain; red rectangle, CFCS; dark yellow rectangle, EHP; brown rectangle, transmembrane domain. The locations of the two ZP3‐specific subdomain elements within the protein's ZP‐C domain, based on the structure of chicken ZP3 (Han et al., 2010), are indicated by a lighter shading. Experimentally supported (Boja et al., 2003; Chalabi et al., 2006; Raj et al., 2017; Zhao et al., 2004) and predicted carbohydrates are depicted as solid and dashed inverted tripods, respectively, with *N*‐glycans colored black and *O*‐glycans colored blue. aa, amino acids, CFCS, consensus furin cleavage site; EHP, external hydrophobic patch; SP, signal peptide; ZP, zona pellucida

The precursor forms of mammalian ZP proteins are composed of a signal peptide (SP), a ZP “domain” module consisting of two immunoglobulin‐like domains (ZP‐N/ZP‐C; Bokhove & Jovine, 2018; Bork & Sander, 1992; Jovine, Qi, Williams, Litscher, & Wassarman, 2004), a consensus furin cleavage site (CFCS; Litscher, Qi, & Wassarman, 1999; Yurewicz, Hibler, Fontenot, Sacco, & Harris, 1993), and a C‐terminal propeptide that contains an external hydrophobic patch (EHP; Jovine et al., 2004), a single‐spanning transmembrane domain (TM; Yurewicz et al., 1993) and a short cytoplasmic tail. The N‐terminal regions of ZP1, ZP2, and ZP4 also contain additional isolated copies of the ZP‐N domain (Callebaut, Mornon, & Monget, 2007; Monné, Han, Schwend, Burendahl, & Jovine, 2008; Nishimura et al., 2019; Raj et al., 2017); moreover, a trefoil (P‐type) domain invariantly precedes the ZP modules of ZP1 and ZP4 (Bork, 1993; Figure 1).

After cotranslational insertion into the endoplasmic reticulum and SP cleavage, ZP precursors undergo a complex folding process that requires formation of intramolecular (ZP1–4) and intermolecular (ZP1) disulfide bonds, as well as *N*‐ and *O*‐glycosylation at multiple sites (Bleil & Wassarman, 1980b; Boja, Hoodbhoy, Fales, & Dean, 2003; Greve & Wassarman, 1985; Greve, Salzmann, Roller, & Wassarman, 1982); notably, in the case of ZP2, *N*‐glycosylation is required for protein secretion (Roller & Wassarman, 1983). The molecules then traffic independently through the secretory pathway as type‐I transmembrane proteins that are eventually packaged into ~2‐µm‐diameter vesicles to reach the plasma membrane (Hoodbhoy et al., 2006; Qi, Williams, & Wassarman, 2002; Qi & Wassarman, 1999). Whereas ZP subunit secretion depends on the EHP (Jovine et al., 2004; Zhao et al., 2003), which constitutes strand G of the ZP‐C β‐sandwich and is thus required for its folding (Han et al., 2010), it does not depend on the TM and the short cytoplasmic tail (Harris, Seid, Fontenot, & Liu, 1999; Jovine et al., 2004; Jovine, Qi, Williams, Litscher, & Wassarman, 2002). However, both of these elements are required for protein incorporation into the ZP of growing oocytes (Jimenez‐Movilla & Dean, 2011; Jovine et al., 2002). Polymerization of the mature ZP subunits depends on their separation from the respective C‐terminal propeptides via C‐terminal cleavage of the precursors in the trans‐Golgi or at the plasma membrane. This proteolytic event is thought to normally target the CFCS (Litscher et al., 1999; Z. Williams & Wassarman, 2001), although alternative processing sites apparently exist that can rescue polymerization of ZP components whose CFSC has been mutated (Zhao, Gold, Ginsberg, Liang, & Dean, 2002). By allowing the EHP to dissociate from the ZP‐C domain, cleavage relieves a polymerization block that prevents premature assembly of ZP subunits and exposes an internal hydrophobic patch that corresponds to ZP‐C β‐strand A (Han et al., 2010; Jovine et al., 2004). This triggers the ZP module‐mediated assembly of the glycoproteins (Jovine et al., 2002), which polymerize into µm‐long filaments. In the mouse, these filaments have a structural repeat of 140–150 Å (Greve & Wassarman, 1985) and show protrusions that likely correspond to the N‐terminal ZP‐N‐containing regions of ZP1 and ZP2 (Monné et al., 2008; Wassarman & Mortillo, 1991). Biochemical and electron microscopy (EM) analysis of ZP filament material treated with reducing agents or chymotrypsin indicated that, by forming intermolecular disulfide bond(s), ZP1 acts as a cross‐linker of individual ZP filaments (Greve & Wassarman, 1985). This suggestion was supported by a recent crystallographic study of the ZP1 cross‐link that, together with mutagenesis experiments, revealed that a single ZP‐N1 domain Cys conserved among species is essential for ZP1 homodimerization in chicken, mouse, and human (Nishimura et al., 2019). Notably, although ZP4 shares the same domain architecture as ZP1 (Figure 1) and their genes are considered paralogous (Conner et al., 2005; Hughes & Barratt, 1999), the cross‐linking function of the two proteins appears to be conserved in chicken (where ZP4 ZP‐N1 makes noncovalent homodimers that can presumably functionally substitute ZP1 in the germinal disk region of the VE) but not in human (where ZP4 ZP‐N1 is a monomer; Nishimura et al., 2019). Because human ZP1 mutations can cause lack of a ZP and sterility (Huang et al., 2014), this suggests that ZP filament cross‐linking plays a much more important role in humans than in mouse (Nishimura et al., 2019). Despite this significant difference, a structurally comparable three‐dimensional network of glycoprotein filaments (5–7‐µm‐thick in mice and up to 13–19‐µm‐thick in humans) surrounds the oocytes of both species.

The ZP has different functions depending on the developmental status of the oocyte/embryo (Wassarman & Litscher, 2018). During oocyte maturation and growth, the ZP maintains the essential connection between the oocyte and the supportive surrounding cells, the cumulus cells (Liu et al., 1996; Rankin et al., 1996). At fertilization, the ZP allows taxon‐specific sperm recognition (Bedford, 1977; Bleil & Wassarman, 1980a; Gwatkin, 1977). After sperm–oocyte fusion, the hardened ZP blocks further sperm binding and protects the embryo during its passage through the female reproductive tract (Braden, Austin, & David, 1954). In the uterus, the mammalian ZP has to be penetrable to allow hatching, which is a requirement for implantation and further embryo development (Cole, 1967). If any of these mechanisms is impaired, no new life will appear; thus, the ZP is essential for mammalian reproduction.

The role of the different ZP subunits in binding sperm at fertilization has been widely debated for decades. On the basis of seminal experiments using purified native material (Bleil & Wassarman, 1980a; Bleil, Greve, & Wassarman, 1988; Florman & Wassarman, 1985) as well as recombinant ZP3 expressed in embryonal carcinoma cells (Kinloch, Mortillo, Stewart, & Wassarman, 1991; Z. Williams, Litscher, Jovine, & Wassarman, 2006), it was suggested that binding of sperm to *O*‐glycans attached to the C‐terminal region of primary receptor ZP3 induces the acrosome reaction, which, in turn, allows acrosome‐reacted sperm to bind to secondary receptor ZP2 (Bleil & Wassarman, 1980a; Bleil et al., 1988). This model was supported by sperm‐binding studies performed using a bacterially‐expressed and refolded N‐terminal fragment of human ZP2 (Tsubamoto et al., 1999), whereas experiments with recombinant ZP proteins refolded from inclusion bodies of baculovirus‐infected insect cells suggested that, in human, acrosome‐intact sperm binds to ZP1, ZP3, and ZP4 (Chakravarty, Suraj, & Gupta, 2005; Ganguly et al., 2010). More recently, it was shown that the acrosome reaction can already occur before direct ZP contact in the upper isthmus of the oviduct (Jin et al., 2011; la Spina et al., 2016); moreover, it was reported that mice lacking the putative C‐terminal *O*‐glycosylation sites of ZP3 are fertile (Gahlay et al., 2010). Together with experiments using recombinant ZP2 expressed in insect cells as well as oocytes with hybrid egg coats containing different combinations of mouse and human ZP proteins (Avella, Baibakov, & Dean, 2014; Baibakov, Boggs, Yauger, Baibakov, & Dean, 2012), this led to the suggestion that sperm of both species bind to the N‐terminus of ZP2 independent of *N*‐ and *O*‐glycosylation (Tokuhiro & Dean, 2018). The currently available data does, however, not exclude that *O*‐glycans positioned elsewhere on ZP3 may underlie its in vitro sperm‐binding activity (Han et al., 2010), and it is possible that the ZP3‐ and ZP2‐centric models of fertilization may simply reflect two alternative pathways of egg–sperm interaction (Wassarman & Litscher, 2018). Nonetheless, all studies agree with the observation that sperm binding to the ZP is abolished upon site‐specific cleavage of ZP2 (Bleil, Beall, & Wassarman, 1981; Gahlay et al., 2010), an event that, in turn, depends on the post‐fertilization exocytosis of the egg's cortical granules (CGs).

## 2 CG EXOCYTOSIS MODIFIES THE ZP AFTER FERTILIZATION

The CGs are secretory organelles of Golgi origin that have a diameter of 0.2–0.6 µm and are anchored in the cortex of unfertilized mammalian oocytes (Austin, 1956; Gulyas, 1980; Liu, 2011). This localization depends on subcortical maternal complex component MATER, and association of the granules with nonmuscular actin motor protein myosin IIA is required for their trafficking to the plasma membrane upon clearance of cortical actin during fertilization and egg activation (Vogt et al., 2019). Fusion of the CGs with the plasma membrane of the oocyte releases their contents into the perivitelline space, the area between the plasma membrane and the ZP (Austin & Braden, 1956). Studies in the hamster showed that, although spontaneous release of CGs already occurs before fertilization, their breakdown gradually increases after gamete fusion and is completed within 7–11 min, that is approximately 3 min after the establishment of blocks to polyspermy at the level of the zona and cell surface (Stewart‐Savage & Bavister, 1991). Because each mammalian oocyte contains thousands of CGs, these are estimated to collectively release about 100 pg protein onto the ZP (Green, 1997). The exocytosis of this material modifies the ZP (Gulyas, 1980) and hardens it by increasing its resistance to proteolytic digestion (Gwatkin, 1964; Smithberg, 1953), as well as changing its mechanical behavior from purely elastic to plastic (Papi et al., 2010). Notably, post‐fertilization hardening of the egg coat is an evolutionarily conserved phenomenon that has also been described in several nonmammalian species, such as sea urchin (Schuel, Wilson, Chen, & Lorand, 1973), the Japanese rice fish/medaka (Nakano, 1956) and *Xenopus* (Grey, Wolf, & Hedrick, 1974).

By containing fewer fenestrations, the hardened mouse ZP matrix is characterized by an increased density (Que et al., 2017) that may alter its enzymatic accessibility by masking protease‐sensitive sites (Green, 1997). Accordingly, transmission and scanning EM studies of human embryos suggest that filament bundles on the inner surface of the ZP are fused together and condensed (Familiari, Heyn, Relucenti, & Sathananthan, 2008). Consistent with these observations, the ZP of embryos becomes thinner (Garside, Loret de Mola, Bucci, Tureck, & Heyner, 1997; Pelletier, Keefe, & Trimarchi, 2004) and stiffer (Drobnis, Andrew, & Katz, 1988; Sun, Nelson, & Greminger, 2005). As a consequence of one or more of these changes, which have been historically combined under the term ZP hardening, penetration of additional sperm through the ZP is prevented (Braden et al., 1954; Inoue & Wolf, 1975; Sato, 1979). The resulting effect on fertilization gave rise to the long‐standing belief that hardening was essential to block polyspermy.

Although ZP hardening was first described decades ago, the biochemical modifications underlying this phenomenon are still mostly unknown; most importantly, it remains unclear if only one process leads to the different characteristics of the hardened ZP, or several processes are involved. Among the possible biochemical processes that could be responsible for hardening, the most important are (a) ovastacin protease‐dependent cleavage of ZP2; (b) deglycosylation of ZP3 and/or other ZP subunits; (c) glycan cross‐linking by lectins; and (d) incorporation of zinc ions into the ZP. A summary of studies that report the effect of treating the ZP with these factors, which are discussed in the following sections, may be found in Table 1.

**Table 1:** Overview of mouse ZP treatments with ZP hardening‐associated factors and the resulting observations

Treatment	Ovastacin/SAS1B	Glycosidases	Lectins	Zinc
Literature	Burkart, Xiong, Baibakov, Jimenez‐Movilla, and Dean (2012); Sachdev et al. (2012); Westphal (2013)	Dolci, Bertolani, Canipari, and Defelici (1991); Miller, Gong, Decker, and Shur (1993)	Dolci et al. (1991); Oikawa, Nicolson, and Yanagimachi (1974); Parkening and Chang (1976)	Que et al. (2017); Tokuhiro and Dean (2018)
Enzymatic resistance	?	Reduced^a^	Increased^a^, ^b^	?
Density	?	?	?	Increased^b^
Filaments fusion and condensation	?	?	?	Yes^b^
Thinning	?	?	?	?
Stiffness	?	?	?	?
Sperm‐binding	Reduced^b^	Reduced^b^	Reduced^b^	Conflicting^a^, ^b^
Sperm penetration	?	?	?	Reduced^b^
Fertilization	Reduced^b^ ^(after treating sperm with ovastacin/SAS1B)^	?	Reduced^b^	?
ZP2 cleavage	Yes^a^	?	?	No^a^, ^b^
ZP protein deglycosylation	?	?	?	?

Abbreviations: ZP, zona pellucida; ?, no data available.

^a^ Performed with isolated ZP.

^b^ Performed with whole oocytes.

### 2.1 ZP2 cleavage

ZP2 accounts for about 40% of the egg coat material and is thus, together with ZP3, a major component of the mouse ZP (Bleil & Wassarman, 1980b). Its mature polypeptide has a predicted molecular weight of 68 kDa (Liang, Chamow, & Dean, 1990) but, due to complex glycosylation, the native protein purified from unfertilized oocytes migrates as a broad band centered around 120 kDa in sodium dodecyl sulfate polyacrylamide gel electrophoresis (SDS‐PAGE; Bleil & Wassarman, 1980b). Interestingly, SDS‐PAGE analysis of ZP material from embryos revealed that, although embryo ZP2 (ZP2f) is indistinguishable from oocyte ZP2 in nonreducing conditions, it migrates at ~90 kDa under reducing conditions (Bleil et al., 1981). This early observation indicated that ZP2 is cleaved following fertilization, and that the products of this processing event remain attached to each other via one or more disulfide bonds. Moreover, it led to the first suggestion that the ZP2 to ZP2f conversion may contribute to ZP hardening, and has an effect on sperm binding by either directly affecting an interaction site with sperm or inducing a global change in the structure of the ZP (Bleil et al., 1981). As further discussed in the following paragraph, post‐fertilization cleavage of ZP2 leading to loss of sperm binding was subsequently observed in *Xenopus* (Gerton & Hedrick, 1986; Tian, Gong, & Lennarz, 1999), where it was also suggested to trigger egg coat hardening (Lindsay & Hedrick, 2004), as well as in human (Bauskin, Franken, Eberspaecher, & Donner, 1999).

By excluding the role of sperm or oviductal components, the finding that ZP2 is also processed when oocytes are activated using calcium ionophore suggested that cleavage is mediated by an egg CG protease (Bleil et al., 1981). This was first characterized in 1989 as a 21–34‐kDa‐enzyme, which could not be blocked by a panel of inhibitors that were used at the time, including EDTA at millimolar concentration (Moller & Wassarman, 1989). Parallel studies in amphibian showed that fertilization of *Xenopus* oocytes induces the release of a salt‐sensitive zinc metalloprotease that cleaves ZP2 homologue gp69/64 at the site ^155^FD|DE^158^ (Tian et al., 1999), corresponding to a [FLM]‐X‐D‐[ED] motif conserved from frog to human (Rankin et al., 2003; Tian et al., 1999). Notably, although its identity remains to be established, the frog protease was found to have the same enzymatic characteristics and substrate specificity of BMP‐1, an astacin‐like metalloprotease (Lindsay & Hedrick, 1989, 2004). Further evidence that members of the zinc‐dependent astacin protease family play an important role in egg coat hardening came from studies of alveolin, an oocyte‐specific enzyme of medaka. In this species, alveolin accumulates into CGs as a proenzyme of 50 kDa that, after processing by a serine protease, is released as an active species of 21.5 kDa at the time of CG breakdown. This form of alveolin hydrolyzes the N‐terminal Pro‐Gln‐X repetitive region of ZPB (a major ZP1‐like component of the egg coat), triggering it's transglutaminase‐dependent intermolecular cross‐linking to ZPC (the medaka homolog of ZP3) and, thus, egg coat hardening (Iuchi, Ha, & Matsuda, 1995; Shibata et al., 2000; 2012).

**Figure 2:**
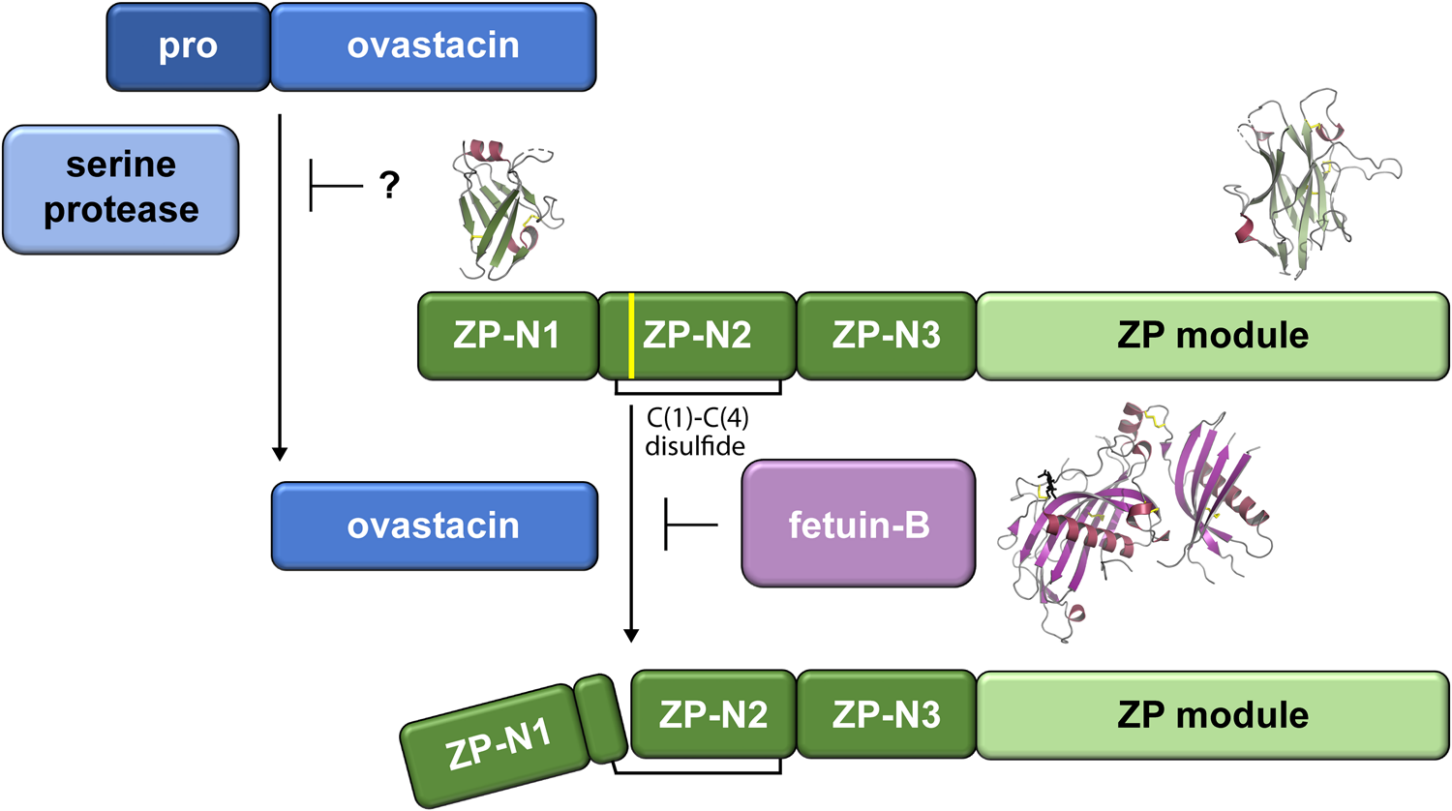
Scheme of the pathway regulating the cleavage of mammalian ZP2. Activation of pro‐ovastacin by a serine protease triggers site‐specific cleavage of ZP2, yielding two protein fragments that remain covalently attached via the predicted C_1_–C_4_ disulfide bond of ZP2 ZP‐N2 (indicated by a black bracket). ZP2 cleavage inactivates the sperm‐binding activity of the ZP as well as increases its resistance to α‐chymotrypsin digestion. Premature processing of ZP2 is counteracted by serum glycoprotein fetuin‐B, which inhibits ovastacin and thus, ZP2 cleavage. Structural information is available for ZP2 ZP‐N1 (PDB ID 5II6), ZP2 ZP‐C (PDB ID 5BUP), and fetuin‐B (PDB ID 6HPV). Note that in this figure, as well as in Figure 3, the ZP module is represented by a single rounded rectangle

Highlighting the evolutionary conservation of the post‐fertilization cleavage of vertebrate egg coat proteins, in 2012 it was suggested that zinc metalloprotease ovastacin mediates the processing of ZP2 in the mouse (Burkart et al., 2012). First identified as a putative mammalian hatching enzyme (Quesada, Sánchez, Alvarez, & López‐Otín, 2004), ovastacin contains a unique heptapeptide motif that ensures its localization in the CGs as a proenzyme of 44 kDa (Burkart et al., 2012; Xiong, Zhao, Beall, Sadusky, & Dean, 2017); following oocyte activation, the protein is released as an active enzyme of 29 kDa that lacks the propeptide as well as a C‐terminal fragment (Körschgen et al., 2017). On the basis of in vitro cleavage studies with plasminogen that was either activated by trypsin or combined with tissue plasminogen activator (t‐PA; Karmilin et al., 2019)—a molecule that is also found in the oocyte CGs (Huarte, Belin, & Vassalli, 1985)—it is thought that in vivo maturation of ovastacin depends on the action of one or more serine proteases (Karmilin et al., 2019; Figure 2).

The mouse ZP2 sequence ^166^LA|DE^169^, located in the second predicted ZP‐N domain of the protein (Monné et al., 2008; ZP2 ZP‐N2; Figures 1 and 2), corresponds to the experimentally determined cleavage sites in frog gp69/64 (Tian et al., 1999) and pig ZP2 (Hasegawa, Koyama, Okazaki, Sugimoto, & Isojima, 1994) and is thus thought to be targeted by murine ovastacin (Burkart et al., 2012). In agreement with this idea, both mutation of the LADE sequence to LGAA and ablation of the *Astl* gene encoding ovastacin abolish ZP2 cleavage, resulting in continued sperm‐binding to two‐cell embryos independent of CG exocytosis (Burkart et al., 2012; Gahlay et al., 2010). Notably, a subsequent study reported that the ZPs of wild‐type MII oocytes are significantly more resistant to α‐chymotrypsin digestion than those of *Astl^−/−^* animals and, unlike ZPs from either MII *Astl* ^−/−^ oocytes or wild‐type GV oocytes, contain a fraction of ZP2 that has been cleaved before fertilization (Körschgen et al., 2017). Although *Astl* ^*−/−*^ mice are less fertile than wild‐type, comparable fertilization rates both in vivo and in vitro indicate that absence of ovastacin has no effect on polyspermy (Burkart et al., 2012; Körschgen et al., 2017; Sachdev et al., 2012); nonetheless, the observed difference in chymotrypsin sensitivity suggests that the cleavage status of ZP2 not only regulates sperm binding but is also directly linked to ZP hardening. Moreover, detection of the C‐terminal domain of ovastacin on the plasma membrane of unfertilized MII oocytes (Körschgen et al., 2017) may explain why ovastacin was also independently described (under the name SAS1B) as an oolemmal binding partner for sperm intra‐acrosomal transmembrane protein SLLP1 (Mandal et al., 2011; Sachdev et al., 2012).

As summarized in Table 1, recombinant mature ovastacin expressed in *Escherichia coli* was found to reduce sperm‐binding to oocytes and fertilization (Sachdev et al., 2012), whereas a partially purified insect cell‐produced version of the enzyme was reported to cleave ZP2 of isolated and washed ZPs (Burkart et al., 2012). Although a possible role of additional factors cannot be completely excluded until it is shown that fully purified recombinant ovastacin can cleave purified recombinant ZP2 in vitro, these findings are in principle compatible with the suggestion that ovastacin processes ZP2 directly.

**Figure 3:**
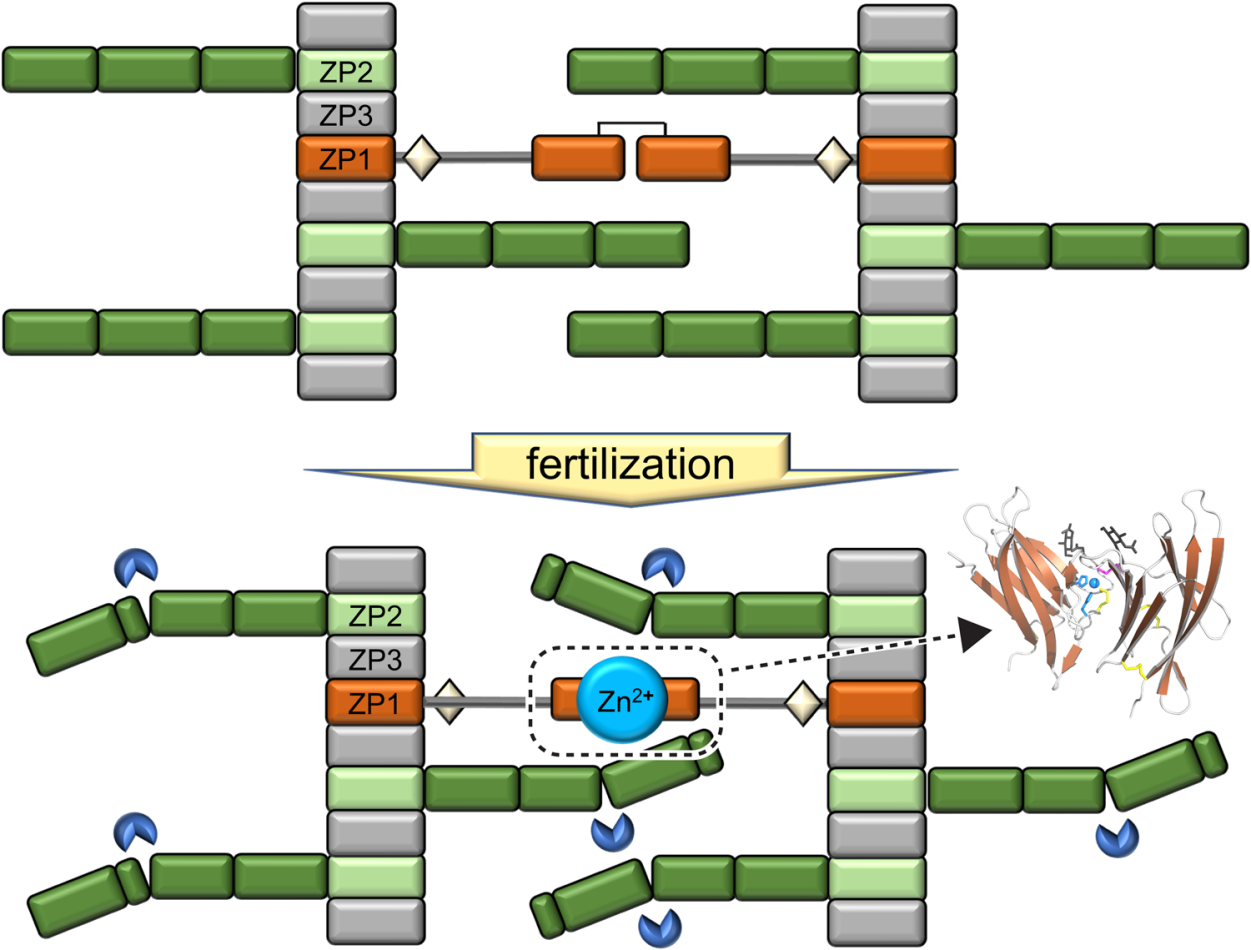
Possible mechanism of mammalian zona pellucida (ZP) hardening. Whereas ovastacin‐dependent cleavage of the ZP‐N2 domain could induce a conformational change of the ZP2 N‐terminus that leads to new protein–protein interactions, binding of zinc ions to ZP1 ZP‐N1 (based on PDB ID 6GF7) may alter the conformation of ZP filament cross‐links. Together with other ZP modifications discussed in the text, these molecular events could cause tighter interaction between adjacent ZP filaments, resulting in compaction of the supramolecular structure of the egg coat. Modified from (Monné & Jovine, 2011). Activated ovastacin is indicated by a blue Pac‐Man‐like symbol

Although high‐resolution structural information is available on both mouse ZP2 ZP‐N1 (Raj et al., 2017) and ZP‐C (Bokhove et al., 2016; Figure 2), it is unknown how cleavage of the protein's ZP‐N2 domain mechanistically induces ZP hardening and blocks sperm‐binding. Together with ZP‐N1 and ZP‐N3 (Figure 1), ZP2 ZP‐N2 is thought to constitute protrusions that project from the ZP filament core (Monné et al., 2008; Wassarman & Mortillo, 1991). Because the LADE cleavage site is relatively close to the region of ZP‐N1 that has been suggested to act as a sperm‐binding site (corresponding to residues 51–149 of mouse ZP2), ZP2 proteolysis could either impair the latter directly or affect it indirectly by causing structural rearrangements that ultimately make it unavailable for sperm. For example, ZP2 cleavage could trigger interaction between the N‐terminal regions of ZP2 subunits belonging to different filaments or, alternatively, allow a cleaved ZP2 molecule on one filament to bind to other ZP subunit(s) on another (Monné & Jovine, 2011; Figure 3). In regard to these possibilities, it would be interesting to know if other fertilization‐triggered ZP modifications, in particular, ZP thinning and increased ZP density, are also impaired in genetically modified mice where ZP2 cleavage is abolished. For the same purpose, as well as to build a more comprehensive picture of ZP hardening, it will also be important to establish whether some of the observations clustered under the term ZP hardening are independent of each other, or instead originate from a common molecular event.

On the basis of the appearance of multiple bands of ~20–35 kDa that reacted with antibodies raised against its ZP‐N1 domain (Burkart et al., 2012; Greenhouse, Castle, & Dean, 1999), it was suggested that post‐fertilization processing of mouse ZP2 is not limited to the LADE sequence, but also involves additional cleavages at positions ^54^DE^55^ and ^127^DD^128^ (Burkart et al., 2012). Considering the disulfide bond pattern of ZP2 ZP‐N1 (Raj et al., 2017), proteolysis at these secondary sites should release a peptide of ~8.7 kDa that would also carry an *N*‐glycan attached to N83 (Boja et al., 2003); as a result, ZP2f would be expected to show a ~10‐kDa shift in SDS‐PAGE migration relative to ZP2, even when analyzed in nonreducing conditions. Consistent with the fact that such a shift was not observed experimentally (Bleil et al., 1981), it was recently shown that the heterogeneity of the N‐terminus of ZP2f is caused by variable *N*‐glycosylation (Tokuhiro & Dean, 2018). Thus, reminiscent of how ZPB is processed by alveolin in medaka, mouse ZP2 is cleaved at a single position by ovastacin.

Of interest in relation to the species‐specificity of ZP2 processing, is the observation that mouse oocytes carrying human ZP2 are subfertile and do not show ZP2 cleavage after fertilization (Rankin et al., 2003; Tokuhiro & Dean, 2018). There could be several reasons for these phenomena. First, the human ZP2 cleavage site (^171^LA|DD^174^) is not identical to that of mouse ZP2, which is also closely followed by a glycosylation site (N172; Boja et al., 2003) that is not conserved in human. Second, the putative sperm‐binding site of ZP2 contains a glycosylation site (N83 in the mouse; Boja et al., 2003), that is conserved in both species. Although the corresponding *N*‐glycan is not required for secretion of mouse ZP2 ZP‐N1 (Raj et al., 2017) or important for sperm–oocyte recognition (Tokuhiro & Dean, 2018), species‐specific glycosylation of the sperm‐binding site of ZP‐N1 in the humanized oocytes may indirectly interfere with processing of the adjacent human ZP‐N2 domain by mouse ovastacin. Third, the C‐terminal part of ovastacin contains an additional domain of ~150 amino acids that differ from sequences found in the corresponding region of other astacins (Quesada et al., 2004). This protein region, which was suggested to play a role in specific substrate recognition (Quesada et al., 2004) and is only 48% identical in sequence between mouse and human (compared to 80% identity for the *N*‐terminal region that precedes it), may influence the ability of mouse ovastacin to process human ZP2. Finally, another explanation could be that the human ZP2 cleavage site is not correctly presented in the context of the nonnative supramolecular structure of the humanized ZP (Rankin et al., 2003); moreover, although the resistance of the human ZP to enzymatic digestion was found to increase over time during oocyte in vitro culture (Schiewe et al., 1995), post‐fertilization ZP hardening in human remains controversial.

Another important aspect to consider is that ZP hardening is not a black and white mechanism, where only a ZP with totally uncleaved ZP2 allows fertilization and a ZP with completely cleaved ZP2 blocks fertilization. Instead, ZP2 cleavage can have many gradations and can also occur, albeit at much lower levels, independent of fertilization (Bleil et al., 1981). To study ZP2 cleavage experimentally, at least 20 oocytes are normally pooled. As a result, it is impossible to assess whether a certain fraction of ZP2 is cleaved in every cell or only a few oocytes contain a much larger proportion of ZP2f. Moreover, as the methods employed for this analysis are destructive, it is impossible to check the ZP2 cleavage status of an oocyte and follow its fertilization at the same time. Despite these limitations, it was found that preovulatory oocytes already contain about 6% ZP2f and ovulated oocytes about 17% (Bleil et al., 1981), without any significant impairment of fertilization success (Dodson, Minhas, Curtis, Palmer, & Robertson, 1989; Körschgen et al., 2017). Furthermore, the level of ZP2f in the ZP of ovulated oocytes increases as a function of time after ovulation or in vitro culture, independent of fertilization (Aonuma, Okabe, Kawai, & Kawaguchi, 1978; Bleil et al., 1981; Dodson et al., 1989; Downs et al., 1986; Gulyas, 1980). Thus, fertilization‐independent ZP2 cleavage appears to be a creeping process that limits the time window for sperm penetration. Because prolonged in vitro culture can decrease the fertilization rate by increasing ZP hardening, this phenomenon is especially crucial when assisted reproductive techniques are used. Under these circumstances, serum glycoprotein fetuin‐B, a cystatin superfamily metalloprotease inhibitor that is essential for mouse fertility (Dietzel et al., 2013), was shown to maintain in vitro fertilization by counteracting ovastacin‐mediated cleavage of ZP2 (Dietzel, Floehr, van de Leur, Weiskirchen, & Jahnen‐Dechent, 2017; Dietzel et al., 2013; Floehr et al., 2016). A recent crystallographic study of fetuin‐B and its complex with crayfish astacin, a nonphysiological binding partner that resembles ovastacin, suggested that the function of the inhibitor depends on a rigid disulfide‐linked CPDCP motif located between its two cystatin‐type modules (Cuppari et al., 2019; Figure 2).

Activated oocytes and two‐cell embryos do not show complete ZP2 cleavage. Instead, only 60–70% of ZP2 is converted to ZP2f (Bleil et al., 1981), but this is sufficient to block sperm‐binding. ZP2 cleavage was initially thought to be essential for blocking polyspermy; however, this process takes between 30 min and several hours depending on the experimental conditions (Baibakov, Gauthier, Talbot, Rankin, & Dean, 2007; Tokuhiro & Dean, 2018), whereas the ZP block to polyspermy is established approximately 5 min after fertilization in the mouse (Sato, 1979). Furthermore, different knockout mouse models showed that impairment of ZP2 cleavage reduces fertility only partially, indicating that ZP2 processing is not essential to ensure monospermic fertilization and further successful development (Burkart et al., 2012; Gahlay et al., 2010; Rankin et al., 2003; Sachdev et al., 2012). The subfertility of these genetically modified female mice is likely to be caused by precocious ZP hatching and early embryonic loss (Burkart et al., 2012; Floehr, Dietzel, Schmitz, Chappell, & Jahnen‐Dechent, 2017; Winuthayanon et al., 2015) and there is no evidence of abnormal sperm accumulation in the perivitelline space of their oocytes (Gahlay et al., 2010; Rankin et al., 2003; Tokuhiro & Dean, 2018). The latter observation suggests the existence of a ZP2‐independent block to sperm penetration, which appears to depend on ovastacin's enzymatic activity based on the presence of supernumerary sperm in the perivitelline space of ovastacin‐deficient oocytes, as well as oocytes expressing ovastacin with an active‐site mutation (Tokuhiro & Dean, 2018). These observations led to the theory that first monospermic fertilization is ensured via a mechanism that does not depend on ZP2, and then cleavage of the latter induces structural rearrangements of the ZP that are essential for protecting the embryo until implantation.

### 2.2 ZP3 deglycosylation

Mammalian ZP proteins are heavily glycosylated (Bleil & Wassarman, 1980b; Boja et al., 2003) and several early observations suggested the role of ZP glycans in egg–sperm interaction and egg coat hardening.

Consistent with the knowledge that sperm binds to eggs but not to embryos (Gwatkin, 1977; Yanagimachi, 1981), ZP3 isolated from unfertilized oocytes, but not from two‐cell embryos, was shown to compete with sperm‐binding (Bleil & Wassarman, 1980a). Partially purified ZP3 *O*‐glycans were found to recapitulate the activity of the glycoprotein and, whereas *N*‐deglycosylated ZP3 was fully active, removal of *O*‐glycans from the protein rendered it as inactive as ZP3 purified from two‐cell embryos (ZP3f; Florman & Wassarman, 1985). Considering that neither ZP1 nor ZP2 could compete sperm binding to the ZP, these findings led to a model according to which, in unfertilized mouse oocytes, ZP3 acts as primary sperm receptor by presenting one or more functionally active *O*‐glycan chains to sperm. Following fertilization, a yet‐to‐be‐identified CG glycosidase then modifies these *O*‐linked carbohydrates to abolish the glycoprotein's activity (Bleil & Wassarman, 1980a; Florman & Wassarman, 1985).

Several findings have been reported through the years, that are seemingly at odds with the ZP3 *O*‐glycan‐centric model of fertilization. For example, treatment of isolated ZPs with an exo‐glycosidase mixture from a sea snail was found to inhibit ZP hardening rather than inducing it (Dolci et al., 1991; Table 1); however, the components of this preparation were not defined and it is unclear if they could even digest *O*‐glycans. Similarly, whereas galactose (Gal) residues at the nonreducing terminus of *O*‐glycans were reported to be essential for mouse ZP3 activity (Bleil & Wassarman, 1988), the terminal glycosyltransferase responsible for adding this type of residue was found not to be expressed in human, apes, and old‐world monkeys (Joziasse, Shaper, Jabs, & Shaper, 1991; Prasad et al., 2000). On the contrary, other studies suggested that *N*‐acetylglucosamine (GlcNAc) residues, rather than galactose, mediate the function of mouse ZP3 by being recognized by sperm surface Gal‐transferase (Miller, Macek, & Shur, 1992); consistent with this possibility, the enzyme catalyzing the hydrolysis of terminal GlcNAc, *N*‐acetylglucosaminidase, was detected in the CGs (Miller et al., 1993). Although the biological relevance of these studies was challenged by reports showing that genetically modified mice lacking complex *O*‐ and *N*‐glycans terminating in Gal or GlcNAc are fertile (Shi et al., 2004; Thall, Malý, & Lowe, 1995; S. A. Williams, Xia, Cummings, McEver, & Stanley, 2007), it remains striking that inhibition of CG *N*‐acetylglucosaminidase leads to continued sperm binding to the ZP after oocyte activation, without impairing ZP2 cleavage (Miller et al., 1993). This clearly implies that enzymatic removal of GlcNAc contributes to the block to polyspermy at the level of the ZP (Table 1), suggesting that the function of *N*‐acetylglucosaminidase, as well as additional glycosidases found in CG exudates (Miller et al., 1993), would be worth further investigation.

Not only the exact chemical nature but also the location of the functional *O*‐glycans of ZP3 has been questioned. This is because, although the number of active ZP3 molecules in the mouse ZP does not seem to be crucial (Liu, Litscher, & Wassarman, 1995), in vitro experiments with recombinant proteins produced in embryonal carcinoma (EC) cells strongly suggested that *O*‐glycosylation of ZP3 S332 and S334 (encoded by *Zp3* exon 7) was essential for its sperm‐binding function (Chen, Litscher, & Wassarman, 1998; Z. Williams et al., 2006). However, mass spectrometric analysis of native mouse ZP3 did not detect *O*‐glycosylation at either position (Boja et al., 2003; Chalabi et al., 2006), and neither sperm‐binding nor fertility is impaired in mice where wild‐type ZP3 is replaced by a ^329^SNSSSS^334^ → ^329^ANVGAA^334^ mutant (Gahlay et al., 2010). A finding that may reconcile these apparently contrasting observations came from the crystal structure of a chicken homolog of ZP3 (Han et al., 2010), which revealed the presence of a single *O*‐glycan attached to T168, a position located relatively close in space to the C‐terminal region of the molecule that is encoded by exon 7 in the mouse. Notably, mutation of the T168 *O*‐glycosylation site significantly reduces the sperm‐binding activity of recombinant chicken ZP3 (Han et al., 2010). Moreover, this site “1” lies within a highly conserved PTWXPF motif of ZP3 which, together with a very closely spaced region (“site 2”), is also *O*‐glycosylated in both native mouse ZP3 and human ZP3 from either transgenic mice or stably transfected mammalian cells (Boja et al., 2003; Chalabi et al., 2006; Zhao et al., 2004). Finally, the N‐terminal fragment that precedes the ZP‐N domain of ZP3, as well residues within β‐strands E and F of the ZP‐C domain (one of which lies relatively close to the aforementioned “site 1”), were shown to also be *O*‐glycosylated in native mouse ZP3 (Boja et al., 2003) or recombinant human ZP3 (Zhao et al., 2004), respectively. Taken together, these observations suggest that the physiological sites carrying the bioactive *O*‐glycans of mouse ZP3 are in fact different from S332 and S334, although preferential modification of the latter may confer the same function to recombinant protein produced in EC cells.

As mentioned above, native mouse ZP3 is *N*‐glycosylated at five positions (Boja et al., 2003; Figure 1) but these glycans are neither required for intracellular trafficking and incorporation into the ZP (Hoodbhoy et al., 2006; Roller & Wassarman, 1983) nor important for gamete interaction (Florman & Wassarman, 1985). On the contrary, as also discussed above, *N*‐glycosylation is necessary for secretion of site ZP2 (Roller & Wassarman, 1983), which also carries an *N*‐glycan attached to N83 within its putative sperm‐binding site (Boja et al., 2003; Raj et al., 2017); however, this carbohydrate is not essential for gamete recognition (Tokuhiro & Dean, 2018), although its possible involvement in ZP hardening remains to be investigated. In contrast with these findings on mouse fertilization, residues such as mannose, fucose, GlcNAc, and the sialyl‐Lewis^X^ sequence [NeuAcα2–3Galβ1–4(Fucα1–3)GlcNAc] of *N*‐ and *O*‐glycans have been suggested to play a role in human egg–sperm binding (Miranda et al., 1997; Oehninger, Patankar, Seppala, & Clark, 2009; Pang et al., 2011). The importance of these chemical moieties may explain the different sperm‐binding properties of affinity‐purified native human ZP proteins (Chiu et al., 2008) compared to those of recombinant counterparts obtained from refolding of insect cell‐ or *E. coli*‐expressed material (which either carried nonnative glycans or lacked glycosylation altogether, respectively; Chakravarty, Kadunganattil, Bansal, Sharma, & Gupta, 2008). At the same time, gamete recognition clearly appears to depend on *N*‐ rather than *O*‐glycans in the pig (Noguchi, Hatanaka, Tobita, & Nakano, 1992; Yonezawa, Aoki, Hatanaka, & Nakano, 1995), and a recent crystallographic analysis of chicken ZP1‐N1 homodimers (Nishimura et al., 2019) suggested that core fucosylation of a conserved *N*‐glycan that immediately precedes the intermolecular disulfide of the protein may favor a specific conformation of the ZP filament cross‐links. This raises the possibility that post‐fertilization defucosylation of ZP1 contributes to hardening of the egg coat (Nishimura et al., 2019).

On the basis of the information summarized so far, it is clear that the presently available data cannot completely rule out the original suggestion that ZP protein glycosylation plays an important role in the interaction between the egg coat and sperm and, consequently, the mechanisms underlying the block to polyspermy and ZP hardening. However, the details of this involvement may differ significantly from what was initially envisaged, as well as vary between species more than it was originally appreciated. Additional studies should thus be undertaken to provide a definitive answer to these questions, in particular with relation to a possible role of the other glycosylation sites of ZP3. At the same time, it is important to consider that glycosylation‐independent recognition of ZP2 and sugar‐mediated binding to ZP3 may not necessarily be mutually exclusive, but rather contribute to a common “hybrid” mechanism (Visconti & Florman, 2010) or constitute alternative ways in which sperm can productively interact with the ZP to accomplish fertilization (Wassarman & Litscher, 2018).

### 2.3 ZP glycan cross‐linking by lectins

As already discussed, proteolytic processing of ZP2 may trigger a structural rearrangement of the ZP2 N‐terminus that could, in turn, lead to tighter filament interaction and, thus, the observed increase in ZP density and other mechanical changes (Monné & Jovine, 2011). Like other possible enzymatic modifications of the egg coat, such a mechanism would be consistent with the relatively small amount of material released by the cortical reaction (Green, 1997); however, ZP2 cleavage cannot intrinsically account for the early zona block to sperm penetration, which appears to be ZP2‐independent.

Considering that the ZP of different mammals has been suggested to have a multilaminar structure (Dandekar & Talbot, 1992; Keefe, Tran, Pellegrini, & Oldenbourg, 1997; Pelletier et al., 2004) and that modification of a thin layer of the egg coat may be sufficient to block sperm penetration, it is possible that the CGs contain enough material to increase ZP stiffness by mechanisms that do not involve enzymes. By binding to one or more ZP proteins, such putative diffusible CG factor(s) may induce conformational changes of the ZP that predate the postulated effect of ZP2 cleavage. Alternatively, CG‐derived molecules could tighten the structure of the ZP by mediating multiple weak, noncovalent interactions between subunits of adjacent ZP filaments. The reversibility of such a mechanism, which may not be easily detectable by SDS‐PAGE if it was only limited to a small fraction of the ZP (and thus, involve relatively few titrating molecules), may be of advantage during hatching, where the ZP needs to be penetrable by the embryo in order for the latter to implant into the uterus.

Candidate molecules that could potentially cross‐link egg coat proteins non‐covalently are lectins released upon CG exocytosis (Chamow & Hedrick, 1986; Dong, Yang, Yang, Zhang, & Gui, 2004; Muñoz‐Gotera, Hernández‐González, Mendoza‐Hernández, Contreras, & Mújica, 2001). Consistent with this possibility, incubation of oocytes with lectins was shown to inhibit fertilization by blocking sperm binding, as well as increase ZP resistance against enzymatic digestion (Dolci et al., 1991; Oikawa et al., 1974; Parkening & Chang, 1976), two markers of ZP hardening (Table 1). As mentioned above, ZP hardening could, on the contrary, be counteracted by incubating isolated ZPs with an exo‐glycosidase mixture (Dolci et al., 1991).

### 2.4 Zinc sparks

The finding that, upon activation, the mammalian oocyte releases zinc in bursts known as “zinc sparks” (Duncan et al., 2016; Kim et al., 2011; Zhang, Duncan, Que, O'Halloran, & Woodruff, 2016) led to the suggestion that this element also plays an important role in blocking polyspermy (Que et al., 2017). As a result of between 1 and 5 zinc spark events that start a few minutes after sperm has penetrated of the ZP, the activated oocyte loses about 12 billion zinc atoms during the first 90 min of activation (Kim et al., 2011; Que et al., 2015; Zhang et al., 2016) and the Zn^2+^ content in the ZP increases by approximately 300% (Que et al., 2017). Remarkably, electron microscopy analysis revealed that zinc exposure increased the density of the ZP and the thickness of its fibrils. These changes in overall ZP architecture were similar to those observed upon egg activation and resulted in decreased sperm binding to Zn^2+^‐exposed ZPs (Que et al., 2017). Together with a recent report that zinc also reduces sperm penetration, independent of ZP2 cleavage (Tokuhiro & Dean, 2018), these findings identify Zn^2+^ as a significant player in the process of ZP hardening (Table 1). Possibly explaining why this phenomenon was not discovered earlier, the zinc release‐associated block to sperm penetration of the ZP covers the time window that is needed to cleave ZP2 (Baibakov et al., 2007; Tokuhiro & Dean, 2018) but only lasts about 9 hr from egg activation, that is, it is lost by the two‐cell embryo stage (Tokuhiro & Dean, 2018).

How could zinc mechanistically affect the architecture of the ZP? Recent structural studies of the N‐terminal domain of ZP1 that cross‐links egg coat filaments revealed that this contains a conserved Zn^2+^‐binding site that does not have enough affinity to scavenge the ion from the cell culture medium, but binds it at concentrations comparable to those liberated by the zinc sparks. Notably, the site lies at the interface between cross‐linked ZP1 moieties and, independently of the presence of zinc itself, mutation of its two Zn^2+^‐binding His in the context of human ZP1 ZP‐N1 severely reduced the protein's ability to form cross‐links in vitro (Nishimura et al., 2019). This finding led to the hypothesis that in addition to possibly bridging other ZP subunits that expose clusters of His, Asp, and Glu residues (Que et al., 2017), zinc ions released by the activated oocyte may modulate the architecture of the ZP by altering the conformation of its ZP1 cross‐links (Nishimura et al., 2019; Figure 3).

The observation that the majority of zinc in the egg is found within the CGs (Kim et al., 2011; Tokuhiro & Dean, 2018), together with the fact that—like all astacins—ovastacin is a zinc‐dependent endopeptidase (Quesada et al., 2004), immediately raised the possibility of a link between the two. Interestingly, it was recently reported that inactivation of the *Astl* gene or its replacement with a variant encoding an active‐site mutant of ovastacin disrupts or alters the CG localization of zinc, respectively. Most importantly, although this study could not detect Zn^2+^‐induced changes of the ZP structure (probably due to the fact that it relied on confocal rather than electron microscopy; Table 1), it showed that zinc exocytosed from the oocyte can abolish penetration of the ZP by inhibiting sperm motility (Tokuhiro & Dean, 2018). Thus, the currently available data suggest that—in addition to allowing resumption of the meiotic cycle (Kim, Vogt, O'Halloran, & Woodruff, 2010)—zinc sparks significantly reduce the ability of both egg and sperm to interact with each other following the first gamete fusion event.

## 3 OTHER FACTORS ASSOCIATED WITH ZP HARDENING

In external fertilizers, covalent cross‐linking of egg coat proteins after fertilization is a common molecular strategy for ensuring the physical protection of the embryo. For example, in addition to the aforementioned transglutaminase activity of fish eggs (Iuchi et al., 1995), an ovoperoxidase released from CGs catalyzes the formation of tyrosine cross‐links that convert the sea urchin VE into a hardened fertilization envelope (Foerder & Shapiro, 1977; Hall, 1978; Veron, Foerder, Eddy, & Shapiro, 1977). Notably, ovoperoxidase activity has also been detected in mouse oocytes activated with calcium ionophore (Gulyas & Schmell, 1980) and ZP hardening was found to be inhibited by compounds that target peroxidases (Schmell & Gulyas, 1980). However, SDS‐PAGE analysis of the ZPs of fertilized or artificially activated mouse oocytes showed no evidence of intermolecular cross‐linking, apart from the ZP1 homodimers that are also found in unfertilized oocytes (Bleil & Wassarman, 1980b; Bleil et al., 1981). Thus, regardless of what is the function of the ovoperoxidase activity of CGs, this does not seem to contribute to post‐fertilization modification of the ZP.

Another CG component that was found to partially associate with the mammalian egg coat after fertilization is peptidylarginine deiminase (PAD)/p75 (Pierce, Siebert, Kopf, Schultz, & Calarco, 1990). Although the possible biological relevance of this interaction remains to be established, a different fraction of PAD molecules remains plasma‐membrane‐associated until the blastocyst stage, suggesting that the protein plays a role in preimplantation development (Liu et al., 2005).

Beside factors released from CGs, oviductal modulators can also bind to the ZP and affect its biological function. For example, prefertilization association of oviduct specific glycoprotein (OVGP1)/oviductin with the ZP was shown to modify its resistance to proteolysis and modulate its sperm‐binding activity, thus controlling polyspermy. Consistent with the observation that different homologues of OVGP1 have variable effects on fertilization, the C‐terminal region of OVGP1 differs among species and regulates the extent to which the protein can penetrate the ZP, in addition to being required for OVGP1 endocytosis into the egg (Algarra et al., 2016; Coy et al., 2008).

## 4 CONCLUSIONS AND FUTURE DIRECTIONS

The concept of “fast” and “slow” blocks to polyspermy reflects the observation that, with the exception of species showing physiological polyspermy, the mechanisms that counteract fertilization by more than one sperm in non‐mammals are generally not only spatially (plasma membrane vs. egg coat) but also temporally distinct. In mammals, on the contrary, there seems to be only a spatial distinction because the membrane and ZP blocks to polyspermy are established at approximately the same time (Gardner & Evans, 2006; Stewart‐Savage & Bavister, 1991). Moreover, with the notable addition of a possible role for rapid Juno shedding after fertilization (Bianchi, Doe, Goulding, & Wright, 2014), the basis of the membrane block to polyspermy—which requires sperm fusion but is neither electrically mediated nor dependent on the contents of the CGs (Horvath, Kellom, Caulfield, & Boldt, 1993; Jaffe, Sharp, & Wolf, 1983)—remains largely unclear (Gardner & Evans, 2006). In comparison, it is by now established that post‐fertilization cleavage of ZP2 induces a definitive block to polyspermy at the level of the egg coat (Bleil et al., 1981; Gahlay et al., 2010). However, the molecular mechanism underlying this block is unknown and we do not appreciate how ZP2 cleavage, as well as other ZP modifications that also influence sperm binding, are connected with the phenomena that are grouped under the term “egg coat hardening.” The recent suggestion that zinc sparks hinder sperm penetration during the time required to cleave ZP2 (Tokuhiro & Dean, 2018) is a step in this direction, but also, in this case, several important aspects, such as the reason why CG localization of zinc is lost in the absence of ovastacin, remain to be explored.

Clearly, elucidating the mechanism(s) that are responsible for the ZP block to polyspermy and its connection with hardening is intrinsically linked to answering the decade‐long quest for the molecular basis of mammalian egg–sperm recognition. The working model of how this essential biological process is mediated has significantly changed through the years, going from the idea that ZP3 and ZP2 act as primary and secondary sperm receptors (Bleil & Wassarman, 1980a; Bleil et al., 1988), to the suggestion that sperm interaction with the ZP does not depend on a single ZP subunit but rather on the supramolecular structure of the egg coat (Rankin et al., 2003), to the more recent hypothesis that the ZP‐N1 domain of ZP2 plays by itself a central role in gamete recognition by physically interacting with (yet‐to‐be identified) counterpart molecule(s) on sperm (Avella et al., 2014). As mentioned above, the observations that led to these different models may not necessarily be irreconcilable; moreover, regardless of what the ultimate answer will turn out to be, it is clear that the architecture of the ZP must be intimately linked to the changes that lead to hardening. In relation to these considerations, it will be essential to gain much more detailed information on the structure of the egg coat than what is currently known. Obtaining this knowledge will not only depend on further X‐ray crystallographic studies of isolated ZP subunits or domains thereof, an approach that already yielded precious information on ZP1 (Nishimura et al., 2019), ZP2 (Bokhove et al., 2016), and ZP3 (Han et al., 2010; Monné et al., 2008), as well as a first example of how sperm recognizes the egg coat in an invertebrate model of fertilization (Raj et al., 2017), but also on cryo‐electron microscopy studies of the ZP matrix. Determining how ZP subunits interact with each other to form filaments, for example, will give us a much more precise picture of which regions of the molecules and associated glycans are exposed in the context of the ZP and thus in principle available for interaction with sperm, as well as binding events/modifications connected with hardening. This knowledge should, in turn, allow us to better interpret the results of the many biochemical studies of these processes that, for experimental reasons, could only be carried out using isolated ZP subunits.

Concerning hardening, it is becoming increasingly clear that what has been historically considered as a single event is instead a complex phenomenon consisting of several processes, which can either occur independently from each other in parallel or be interlinked. For example, ovastacin cleavage of ZP2 may inactivate its sperm‐binding site whereas ZP protein cross‐linking could underlie the post‐fertilization increase in ZP stiffness and resistance against enzymatic digestion. On the contrary, if cleaved ZP2 forms new intermolecular contacts within the ZP that mask its sperm‐binding site, the aforementioned functional and mechanical changes would reflect different aspects of the same molecular event.

Finally, it is also important to consider how ZP hardening influences further embryo development. It has already been reported that insufficient hardening leads to early embryonic loss (Gahlay et al., 2010; Winuthayanon et al., 2015), but whether there are any consequences of excessive hardening, especially in relation to embryo hatching, remains unclear. By creating a situation that is structurally very different from that of oocytes with an abnormally thicker ZP, a feature that does not appear to impair hatching (Syrkasheva et al., 2017), super‐hardening of the egg coat could potentially have a significant effect on the success rate of assisted reproduction in humans.

## CONFLICT OF INTERESTS

The authors declare that there are no conflict of interests.
